# Current perspectives and practices of newborn vitamin K administration in low and middle income countries

**DOI:** 10.2147/RRN.S154652

**Published:** 2018-04-05

**Authors:** Patricia S Coffey, Emily Gerth-Guyette

**Affiliations:** PATH, Seattle, WA, USA

**Keywords:** newborn, vitamin K, prophylaxis, LMIC

## Abstract

**Background:**

Vitamin K prophylaxis can prevent vitamin K deficiency bleeding (VKDB), and current global recommendations support universal prophylactic use in newborns. Data about access to and use of vitamin K in low and middle income countries (LMIC) are scarce. To address this gap, we explored current perspectives and practices of newborn vitamin K administration in LMIC in order to better understand the barriers to more widespread coverage of this lifesaving preventative treatment.

**Methods:**

We conducted an online survey of stakeholders involved in newborn health. We sent the survey via e-mail to 109 individuals who were based primarily in LMIC and 23 responses were received, resulting in a response rate of 21%. Respondents were generally health or development professionals from sub-Saharan Africa and Asia.

**Results:**

Incidence rates at the country level were mostly unknown or not supported by adequate data. Many respondents (17/23) indicated that vitamin K prophylaxis is included in their national newborn care guidelines and policies, while 12 respondents indicated that administration at birth was widely practiced. Around half of respondents reported that health workers were trained in the diagnosis and treatment of VKDB. The most frequently cited barriers to more widespread vitamin K prophylaxis were (in rank order) high rates of home birth (which preclude injections that must be given by skilled health workers), lack of access to and availability of vitamin K, perception that vitamin K prophylactic treatment is not a priority among health workers, lack of vitamin K formulations appropriate for infants, cultural practices suggesting that injection at birth is not acceptable to parents, and vitamin K not being included in national guidelines and policies. There was no consensus as to the ideal formulation, respondents preferring both the current intramuscular (IM) injection and oral formulation. Reported product attributes of IM and oral formulations are summarized.

**Conclusion:**

Prophylactic administration of vitamin K to newborns is relatively well integrated into policy at the global and country levels, but its practice is underutilized. Barriers to use are access, supply chain logistics, provider attitudes, and restrictions on the use of injections by providers at the community level. Technology innovation may offer some promise to mitigate these barriers, although advocacy and health system strengthening might be more likely to yield improved coverage. Further investigation using in-depth bottleneck analysis at the country level could help identify specific health system improvements.

## Background

Vitamin K deficiency bleeding (VKDB) is a rare and potentially dangerous bleeding disorder in an infant in the first hours to months of life. Vitamin K is necessary to regulate normal homeostasis. Without sufficient stores of vitamin K, neonates are at risk of spontaneous bleeding or bruising that can occasionally lead to lethal hemorrhage.^1^ The disease presents in one of the following three ways: early, classic, and late.^2^ Early VKDB occurs within 24 hours of birth and is very rare and usually a consequence of drugs that have been given to the mother (such as anticonvulsants) that interfere with metabolism. Classic VKDB occurs 1–7 days after birth and is known to be caused by inadequate feeding and milk intake. Late VKDB occurs 8 days to 6 months after birth, most often between 3 and 7 weeks in infants who are exclusively breastfed. Common classic VKDB sites include gastrointestinal, umbilical cord, skin, nose, and site of circumcision. Common sites for late VKDB include intracranial and gastrointestinal; late VKDB is also associated with cholestatis or liver dysfunction that prevents nutrient absorption in the infant.^3^,^4^ Newborns are at particular risk of deficiency as vitamin K does not readily pass through the placenta and breast milk is not an adequate source of the vitamin.^5^,^6^ Formula feeding of newborns offers more protection against VKDB, as formulas contain much higher levels of vitamin K, though this ancillary benefit is not significant enough to outweigh the many benefits of exclusive breastfeeding.^7^

Vitamin K prophylaxis can prevent VKDB.^8^ The World Health Organization (WHO) recommends that newborns receive a 1 mg of intramuscular (IM) injection of vitamin K at birth. Evidence from multiple surveillance studies shows that the introduction of vitamin K prophylaxis reduces the incidence of VKDB.^3^ Current recommendations support universal prophylaxis due to the lack of predictors for vitamin K deficiency^9^. Despite these recommendations, coverage of vitamin K prophylactic treatment in low-resource settings is low.^10^ Moreover, VKDB is re-emerging in developed countries due to the increasing rate of exclusive breastfeeding and the increase in parents who refuse injections – including vaccines and vitamin K prophylaxis at birth.^11^ Oral formations are available, but the optimal regimen and associated efficacy of oral doses are not well understood. The most common oral regimen involves three doses of 2 mg each with the first dose given at birth, and the subsequent two doses given over the course of the next 1–3 months.^12^ Oral vitamin K, either single dose or multiple dose, has not been tested in randomized trials for its effect on either classic VKDB or late VKDB. While oral formulations offer an alternative to injections, the available oral dosage form may not be appropriate for newborns.^12^

Ensuring that newborns receive vitamin K is particularly critical in places where access to health care and blood products and transfusions is limited. Anecdotal evidence suggests that an investigation into the barriers preventing access to and widespread use of vitamin K prophylactic treatments in low-resource settings is warranted. To address this gap, we explored the current perspectives and practices of new-born vitamin K administration in low and middle income countries (LMIC) in order to better understand the barriers to more widespread coverage of this lifesaving prophylactic treatment. The primary objectives of this formative research were to 1) understand if VKDB is viewed as a public health need unmet by practitioners and policymakers in LMICs, 2) articulate key barriers to use the current prophylactic vitamin K regimen in LMICs, and 3) explore alternatives to injectable vitamin K.

## Methods

Given the lack of literature on perspectives and practices regarding VKDB, particularly in LMICs, we conducted a formative on-line survey of global stakeholders involved in newborn health. Surveys delivered via online platforms offer advantages and disadvantages to both researchers and participants. Online surveys are cost effective, easy to complete, and allow participants to respond anonymously. However, the response rate for online surveys is on average 10% lower than for other delivery methods, and access is limited.^13^ We developed the self-administered survey based on gaps in existing literature and solicited perspectives from individual stakeholders through both close- and open-ended questions, designed to encourage respondents to share their expert opinions. The survey consisted of 11 questions that covered the following four thematic areas:

- Perspectives on newborn bleeding.
- VKDB prophylaxis practices.
- Availability of vitamin K.
- Barriers to the widespread use of vitamin K.

We distributed the survey electronically to known newborn health experts as well as individuals suggested by respondents who had completed the survey. We placed an emphasis on obtaining feedback from experts and stakeholders currently working in newborn health in LMIC. To maximize participation, we invited participation from various global stakeholder groups including the United Nations Commission on Life Saving Commodities Newborn Technical Resource Team and a large maternal child health and survival project funded by the United States Agency for International Development (USAID).

The data were coded and analyzed using Microsoft Excel 2016 (Redmond, Washington, DC, USA). Frequencies and percentages of different variables were tabulated. In countries with multiple respondents, some responses were contradictory. In these cases, responses are reported individually. Narrative responses to open-ended questions were collated manually and analyzed for thematic content.

The PATH Research Determination Committee reviewed this survey protocol and determined that it is not human subjects research as it does not meet the definition of research provided by the US government (45 CFR 46.102[f]) and the Centers for Disease Control. Respondents consented to participate in the survey by completing the survey. The online survey stated that participation on the part of participants was voluntary and that they could refuse to answer any question. Identifiers linking participants to their responses were removed from the survey prior to data analysis.

## Results

We sent the survey via e-mail to 109 individuals who were based primarily in LMIC, and 23 responses were received, resulting in a response rate of 21%. We received one response from Cameroon, Denmark, Dominican Republic, Ethiopia, Nepal, Nigeria, Papua New Guinea, Philippines, Rwanda, Serbia, South Sudan, Zambia, and Zimbabwe, two responses from India, Malawi, and the USA, and four responses from Kenya. Respondents from the USA were global newborn health experts who have extensive experience working in LMIC environments. The respondent from Denmark based her response on work experience in Namibia. Survey respondents included neonatologists, pediatricians, nurses, and public health officers practicing in LMIC; maternal, child, and newborn health program managers and advisors; and technical newborn health specialists.

### Stakeholder perspectives

**Figure 1:**
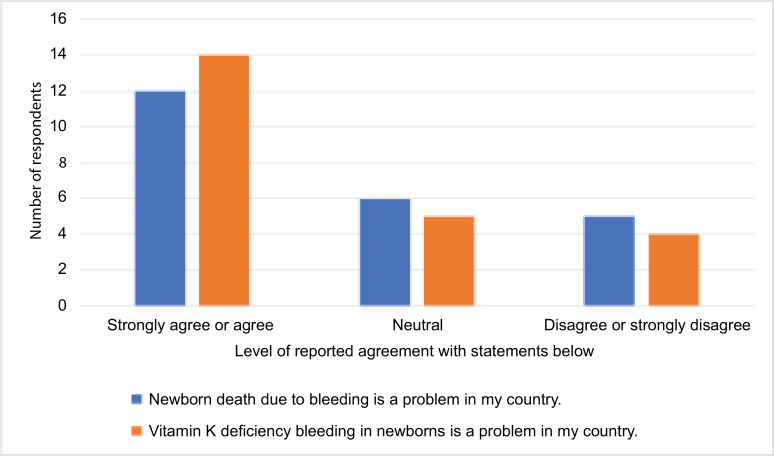
Stakeholder perspectives on the newborn bleeding and vitamin K deficiency bleeding.

Around half of respondents either strongly agreed or agreed that newborn death due to bleeding was a problem in their respective countries, and about two-thirds of respondents either strongly agreed or agreed that VKDB in newborns was a problem (Figure 1).

### Global policies and practices

Incidence rates at the country level were mostly unknown (6/23) or not supported by adequate data (13/23). Many respondents (17/23) indicated that vitamin K prophylaxis is included in their national newborn care guidelines and policies, while 12 respondents indicated that administration at birth was widely practiced. Around half of respondents reported that health workers were trained in the diagnosis and treatment of VKDB. Where vitamin K prophylaxis is administered, it is most often given at the same time as eye ointment and less frequently with the oral polio, hepatitis, and *Bacillus* Calmette Guérin (BCG) vaccines. Table 1 provides an overview of responses by country.

**Table 1:** Global policies and practices by country

Country	Included in guidelines and policies	Administration is widely practiced	Health workers are trained
Cameroon	No	No	No
Dominican Republic	Yes	Yes	Yes
Ethiopia	Yes	No	Yes
India (respondent 1)	No	No	No
India (respondent 2)	Yes	Yes	Yes
Kenya (respondent 1)	Yes	No	No
Kenya (respondent 2)	Yes	Yes	No
Kenya (respondent 3)	Yes	Yes	No
Kenya (respondent 4)	Do not know	No	No
Malawi (respondent 1)	Yes	No	No
Malawi (respondent 2)	Yes	No	Yes
Namibia	Yes	Yes	Yes
Nepal	No	No	No
Nigeria	Do not know	Yes	No
Papua New Guinea	Yes	No	No
Philippines	Yes	Yes	Yes
Rwanda	Yes	Yes	Yes
Serbia	No	Yes	Yes
South Sudan	Yes	No	No
United States (respondent 1)	Yes	Yes	Yes
United States (respondent 2)	Yes	Yes	Yes
Zambia	Yes	No	No
Zimbabwe	Yes	Yes	Yes

Respondents offered explanations as to why vitamin K prophylaxis was not widely practiced. The most frequently cited barriers to more widespread vitamin K prophylaxis are (in rank order): high rates of home birth (which preclude injections that must be given by skilled health workers), lack of access to and availability of vitamin K, perception that vitamin K prophylactic treatment is not a priority among health workers, lack of vitamin K formulations appropriate for infants, cultural practices suggesting that injection at birth is not acceptable to parents, and vitamin K not being included in national guidelines and policies. Respondents further elaborated that health workers are not likely to request that vitamin K is restocked, resulting in a subsequent lack of supply.

### Preferred vitamin K formulations

Of the 12 respondents who specified a formulation of vitamin K, ten respondents indicated that IM injection alone was recommended and two indicated that both injections and oral doses were recommended. Just under half of these respondents indicated that delivery through injection at birth posed a barrier to its use. Most respondents suggested that an oral formulation may convey some benefits over IM injections. The most frequently cited benefits of oral dosages included (in rank order) ease of administration, greater acceptability among health workers and parents, potential to achieve higher rates of coverage, and increased likeliness to be included in national guidelines and policies. Nonetheless, some respondents were more critical of an oral formulation and raised concerns regarding efficacy, swallowing issues, and treatment compliance with a multi-day oral regimen. There was no consensus as to the ideal formulation. Respondents were divided between the current IM injection and the potential of an oral formulation. One respondent suggested that a single oral dose would be ideal, and another indicated that IM injections should be used at facilities and oral doses should be available for home deliveries. Table 2 summarizes the reported attributes of each product formulation.

**Table 2:** Reported product attributes of IM injection vs oral formulation

Type of formulation	Advantages	Disadvantages
IM injection	Single dose Demonstrated efficacy No compliance issues	Requires skilled provider at birth Requires additional administration supplies (such as syringes and needles) Issues of acceptability among caregivers
Oral formulation	Does not require needle- or syringe-skilled provider More acceptable to caregivers Not painful Less expensive	Issues of compliance with multi-day regimen Uncertainly around efficacy and appropriate regimen

**Abbreviation:** IM, intramuscular.

Beyond the improvement of oral vitamin K, respondents identified a number of other ways to improve the coverage of vitamin K prophylaxis. These recommendations included the provision of procurement support, advocacy and awareness raising activities both at global and country levels including vitamin K as a part of essential newborn care training programs, generating better data on incidence rates, and training health workers on the importance of vitamin K. Some specific suggestions are noted below:

“Training of health workers in the administration and sensitizing the community on the importance.” [Health and nutrition program manager in Zambia]

“The Ministry of Health (MoH) should deliberately put forward a policy to procure vitamin K and a memo should be circulated to facilities once the drug is procured demanding all facilities and providers to provide vitamin K.” [Maternal and newborn health expert in Malawi]

“I would encourage the linkage of these efforts with those to disseminate Essential Care for Every Baby, so that there is a more extensive educational framework around the initiative.” [Professor of research in Kenya]

## Discussion

This targeted survey was intended to provide formative information in a neglected area of newborn care rather than definite conclusions. Findings from this survey suggest that awareness and prevention of VKDB is viewed as an unmet public health need in sub-Saharan Africa and Asia. Although about half of the respondents stated that they believed that newborn death due to bleeding and VKDB, in particular, was a problem in their country, global data on the incidence of VKDB are scarce. While some countries participate in national surveillance, these studies are sporadic and outdated.^3,14–16^ Incidence data may also be complicated given that a case of VKDB can only be confirmed as such through laboratory-based blood tests that assess the time it takes blood to clot.^17^

Available evidence, including the results from this study, suggests that vitamin K deficiency in LMIC may be common. For example, in a recent study in Uganda, insufficient levels of vitamin K were found in 33% of mothers and 66% of newborns.^9^ Moreover, modeling of estimated incidence of VKDB in three scenarios in LMIC shows that late VKDB might occur at 1) the same rate as that found in developed country settings (7/100,000), 2) a fourfold increase above developed county estimates due to the relatively higher levels of exclusive breastfeeding and nutritional deprivation (28/100,000), or 3) much higher levels than high-resource settings based on data from a surveillance study at a single hospital in Thailand (72/100,000).^7^

Data from our survey suggest that prophylactic administration of vitamin K to newborns was relatively well integrated into policies at the global and national levels such as in Ethiopia where administration of newborn vitamin K is one of the eight essential steps of newborn care in the Integrated Management of Neonatal and Childhood Illness (IMNCI) package.^18^ Barriers to administration appear to be related more to supply of and access to the drug, cultural perspectives on the appropriateness of injection for newborns, and acceptability of the practice by health providers and caregivers rather than policy alignment. Targeted interventions have demonstrated potential to improve the coverage of vitamin K prophylaxis. In India, a project in 27 USAID-supported districts increased provision of vitamin K to the newborn from 45% to 95% over a 14-month period.^19^ Furthermore, it is encouraging to know that vitamin K administration is clearly positioned as a critical element in the current versions of the Essential Care for Every Baby and Essential Care for Small Babies curricula.^20,21^

The uneven practice of vitamin K administration may be due, in part, to potentially misleading global policy. A minority of respondents stated that vitamin K was to be used only with preterm infants. This may stem from two related and distinct WHO recommendations. The first recommendation states that “All newborns *should* receive a 1.0 mg IM injection of vitamin K at birth.”^10^ The second recommendation specifies that “Neonates requiring surgical procedures, those with birth trauma, preterm newborns, and those exposed in utero to maternal medication known to interfere with vitamin K are at especially high risk of bleeding and *must* be given vitamin K.”^10^ A systematic review of evidence of effectiveness of interventions for preterm birth issues a strong recommendation for postnatal vitamin K use in preterm births.^22^ This lack of clarity around the use indication for preterm vs all infants may be contributing to provider confusion about best practice.

A recent review article highlights the paucity of evidence regarding the efficacy of vitamin K prophylaxis for preventing late VKDB and calls for further investigation into the safety and efficacy of oral formulations.^23^ Oral formulation of vitamin K may have the potential to address some of the barriers to widespread administration of the injectable formulation by increasing acceptability to providers and caregivers. The investment necessary to generate clinical data around efficacy, regimen, and compliance barriers to the use of syringe and needle injections by health workers at the community level.

This study is limited by its small sample size and the delivery of the survey through an online platform. Due to the distribution method via electronic networks and list serves, the survey response rate is uncertain. While low response rates alone do not necessarily indicate a response bias, results cannot be generalized to all country experiences.^24^ Furthermore, data from this study reflect inconsistencies within countries, most likely due to varying practices at different levels of the health system. Additional data regarding current practices of vitamin K prophylaxis and efforts to raise awareness about the importance of newborn vitamin K administration are needed.

## Conclusion

Although prophylactic administration of vitamin K to newborns is relatively well integrated into policy at the global and country levels, its practice appears to be underutilized. Barriers to use span access are supply chain logistics, provider attitude, and restrictions on the use of injections by providers at the community level. Technology innovation may offer some promise to mitigate these barriers, although advocacy and health system strengthening might be more likely to yield improved coverage. Further investigation using in-depth bottleneck analysis at country level could prove helpful in identifying specific health system improvement needs.

## Data sharing statement

The dataset generated and analyzed during the current study is available from the corresponding author on reasonable request.
